# Supplementary material for: Genomic insights into the role of Salmonella Typhi carriers in antimicrobial resistance and typhoid transmission in Urban Kenya
Source: PLoS One. 2025 May 28;20(5):e0321879. doi: 10.1371/journal.pone.0321879 (PMC12118847; doi:10.1371/journal.pone.0321879)
Supplement: S1 Table — (DOCX) [file pone.0321879.s001.docx]

**Supplementary Table 1: Genomic features of *S*. Typhi from individuals living in Mukuru, Nairobi, Kenya**

| **Sample ID** | **Genome size**  **(bp)** | **GC (%)** | **Number of Contigs** | **N50** | **GenBank Accession** |  |
| --- | --- | --- | --- | --- | --- | --- |
| 020127_C4-F5 | 4735688 | 52.1 | 72 | 204167 | JBEUSW000000000 |  |
| 020127_C4_F3 | 4735093 | 52.1 | 77 | 204125 | JBEUSV000000000 |  |
| 020127_C4_F4 | 4735139 | 52.1 | 77 | 204170 | JBEUSU000000000 |  |
| 27737_C1_F2 | 4735283 | 52.1 | 77 | 204170 | JBEUST000000000 |  |
| 20113 | 4832818 | 52.0 | 87 | 204125 | JBEUSS000000000 |  |
| 043198_F4 | 4923312 | 51.8 | 83 | 178823 | JBEUSR000000000 |  |
| 043198_F1 | 4922366 | 51.8 | 84 | 178823 | JBEUSQ000000000 |  |
| 43198B | 4922356 | 51.8 | 88 | 153397 | JBEUSP000000000 |  |
| 43198S | 4922276 | 51.8 | 85 | 161070 | JBEUSO000000000 |  |
| 28946 | 4735038 | 52.1 | 78 | 204125 | JBEUSN000000000 |  |
| 49232 | 4735408 | 52.1 | 73 | 204125 | JBEUSM000000000 |  |
| 28966 | 4735500 | 52.1 | 71 | 204170 | JBEUSL000000000 |  |
| 027750_F2 | 4735677 | 52.1 | 75 | 204125 | JBEUSK000000000 |  |
| 27962 | 4735683 | 52.1 | 76 | 204125 | JBEUSJ000000000 |  |
| 27750B | 4735424 | 52.1 | 73 | 204169 | JBEUSI000000000 |  |
| 27750S | 4735703 | 52.1 | 77 | 204125 | JBEUSH000000000 |  |
| 37806S | 4735481 | 52.1 | 73 | 204125 | JBEUSG000000000 |  |
| 37598B | 4735609 | 52.1 | 73 | 204170 | JBEUSF000000000 |  |
| 37598S | 4735131 | 52.1 | 79 | 204125 | JBEUSE000000000 |  |
| 37623 | 4736541 | 52.1 | 76 | 204125 | JBEUSD000000000 |  |
| 17209 | 4737189 | 52.1 | 75 | 204170 | JBEUSC000000000 |  |
| 27344 | 4735457 | 52.1 | 78 | 204170 | JBEUSB000000000 |  |
