# Supplementary material for: Genomic insights into the role of Salmonella Typhi carriers in antimicrobial resistance and typhoid transmission in Urban Kenya
Source: PLoS One. 2025 May 28;20(5):e0321879. doi: 10.1371/journal.pone.0321879 (PMC12118847; doi:10.1371/journal.pone.0321879)
Supplement: Attachment — Submitted filename: Plos One Response to Reviewers (1) (1).docx [file pone.0321879.s004.docx]

| **PLOS ONE RESPONSES TO REVIEWERS** | | |
| --- | --- | --- |
| **Comment** | **Response** | **Where on Manuscript** |
| **REVIEWER 1** | | |
| The authors should work further on the manuscript so that it concisely recounts the findings and has some good copy editing before submitting it again. | The authors greatly appreciate the reviewer's comments and have taken them into consideration to improve the manuscript. | Throughout the manuscript |
| Comment: Salmonella typhi should be written in italics with the t in lower case: S. typhi. | We appreciate this comment. Only *S* in *S*. Typhi is italicized, per the binomial nomenclature for naming Salmonella species. The Serovar Typhi remains normal and with a capital T. We have made this uniform across the manuscript. We will attach a document supporting this special naming system | Throughout the manuscript |
| Line 66 and others: in “Globally, the TF…”, the word “the” is unnecessary fever in line 69 | Thank you for this comment/suggestion. We have removed’ the’ appearing before Typhoid | Line 70 |
| The introduction is too long and goes into details about gallbladder colonization and other topics that are unnecessary. | Thank you for this comment/suggestion. The introduction has been redone to only include the necessary topics | Throughout the introduction |
| Line 125 What is the definition of a carrier? Any asymptomatic household contact with S. typhi in the stools or rectal swab? | Thank you for this comment/suggestion. A carrier is an individual shedding *S*. Typhi bacteria in their stool without showing typhoid related symptoms. | In line 132-133, we define who contacts are |
| How long did they carry, how many per household? Were the isolates from the cases and their household contacts always exactly the same? | Thank you for this comment/suggestion. The individuals found to be shedding *S*. Typhi, both cases (post treatment for cases) and household contacts were to be followed up for three months to check how long they were shedding, however after three consecutive negative cultures for *S*. Typhi the follow up was stopped;  The individuals recruited per household were at least three per household;  We cannot make the conclusion whether the isolate from the cases and their household contacts were exactly similar, since not all of them were sequenced This has been highlighted as a limitation. | Lines 157-162  Lines 149-151.  Lines 452-444 |
| Figure 1 – the schematic map on the right is impossible to read because the font is too small | Thank you for this comment/suggestion. The map has been removed from the manuscript due to copy right reasons. The sampling sites are now mentioned and described in narration in the manuscript | Lines127-134. |
| Line 134 What is meant by “with/out” | Thank you for this comment/suggestion. This has been revised to with or without. | Line 138 |
| Line 242. Presumably they mean that P values < 0.05 were taken as significant, but this is not stated. | Thank you for this comment/suggestion. This has been stated clearly on the manuscript. | Line 246-250 |
| Line 245 What is meant by the sentence, “S. Typhi isolated from both cases and carriers aged ≥20 years were most prevalent at 52% &65% cases and carriers respectively.”? | Thank you for this comment/suggestion. This has been rephrased to make more sense and have better flow to show the proportions of cases and carriers of *S*. Typhi that were aged ≥20 years were the most abundant. | Lines 256-257 |
| Line 251 THE majority | Thank you for this comment/suggestion. ‘The’ has been removed from appearing before the word majority. | Line 260 |
| Lines 253 – 4. The meaning is not clear. | Thank you for this comment/suggestion. This has been revised. | Lines 257-260 |
| Line 262. 11 (10%) of cases. The “of” is not needed. And it is 10% of what? 11/??, same in the next line: 34/?? This is a recurrent problem: it is not clear what populations these are percentages of. | Thank you for this comment/suggestion. The results here are now expressed as n/N (%) to show the populations these proportions are of. This has been redone across the manuscript and in this section. | Lines 270-275 |
| Line 267 Of what are 15 isolates with nalidixic acid resistance 13%? This is a problem throughout the manuscript | Thank you for this comment/suggestion. This has now been redone to show the denominators of this numbers in order to support the proportions for ease of reference | Lines 276-282 |
| Figure 2 The overall number of cases and controls should be shown in the legend | Thank you for this comment/suggestion. This has now been changed to a table and the overall numbers of cases and carriers has been added. | Line 283-284 |
| Lines 283-5 Lineage 1 is not discussed previously and only mentioned in the discussion line 392-3, so in this location in the results it is unknown what is being referenced. | Thank you for this comment/suggestion. This statement now has proper referencing from the results section. | Lines 292-296 |
| Line 302-3 are unclear | Thank you for this comment/suggestion. This has been revised and the entire section. | Lines 305-308 |
| Figure 3 is odd and the format makes it difficult to understand, in addition, the in the purple boxes the background is too dark for the dark letters to be legible. | Thank you for this comment/suggestion. Figure 3 is now figure 2 and has been revised to another format for ease of reference, | Lines 313-314 |
| Line 311 “2(95) in carriers”. What is 95? The “in” is unnecessary. | Thank you for this comment/suggestion. This section has been revised. The 95% in the bracket should have been a 9% that was a typographical error. The in before carriers was also removed and the entire section rephrased for better flow | Lines 319-324 |
| The discussion is much too long and rambling. Fluoroquinolone resistance could be treated much more concisely | Thank you for this comment/suggestion. The discussion has been redone to only include what is included in the results and too much details removed from it. | Discussion |
| Lines 411-13. If the AMR genes showed no discrimination, how could transmission be identified? | Thank you for this comment/suggestion. We postulate that transmission can happen in a household and community considering that isolates recovered from among some of the cases and carriers belonged to the same lineage and sub lineage.  We have however included a phylogenetic tree to show the relatedness of our isolates. | -  Lines 301-303 |
| REVIEWER 2 | | |
| Take into consideration making the title more focused and specific. For example: "Genomic Insights into the Role of *Salmonella Typhi* Carriers in Antimicrobial Resistance and Typhoid Transmission in Urban Kenya." | Thank you for this comment/suggestion. The tittle has been revised accordingly as advised. | Lines 1-2 |
| The focus on carriers and AMR profiles in the study should be clearly reflected in the title. | Thank you for this comment/suggestion. This has now been revised to reflect focus on carriers and AMR profiles as well. | Lines 1-2 |
| The primary findings, including the frequency of genotypes and associated AMR patterns, should be outlined in the abstract. Public health recommendations should be included at the end. | Thank you for this comment/suggestion. The abstracts result section has been modified to describe the findings better. | Lines 38-55 |
| Give a one-sentence explanation of the methodology, highlighting AMR characterization and whole genome sequencing. | Thank you for this comment/suggestion. This explanation has now been included in the abstract as guided. | Lines 34-38 |
| Do not repeat information (for example, resistance to first-line antibiotics is mentioned twice). | Thank you for this comment/suggestion. This has been revised accordingly. | - |
| More quantitative information regarding the prevalence of typhoid fever in Kenya and its regional and worldwide distribution should be provided. | Thank you for this comment/suggestion. The above information has now been provided. | 74-76 |
| Explain briefly why disease transmission is most prevalent in urban informal communities. | Thank you for this comment/suggestion. An explanation on typhoid disease transmission being most prevalent in urban informal communities has now been provided on lines… | Lines 77-80 |
| Draw attention to inadequacies in earlier research, especially with relation to carriers' involvement in AMR transmission. | Thank you for this comment/suggestion. inadequacies in earlier research, especially with relation to carriers' involvement in AMR transmission have now been added on lines 88-89. | Lines 91-93 |
| Although the novelty and rationale are not stated clearly, describe how the study fills in knowledge gaps about the role of carriers in AMR. | A description of how the study fills in knowledge gaps about the role of carriers in AMR has now been included. | Lines 116-120 |
| The introduction briefly mentions AMR-associated genotypes. Expound on why genotype 4.3.1 (H58) is significant in the Kenyan context. | Thank you for this comment/suggestion. An expansion has now been provided as to why genotype 4.3.1 (H58) is significant in the Kenyan context on lines 101-102 | Lines 105-106 |
| Although the longitudinal design is obvious, the rationale behind the frequency of sample collection and follow-up periods should be given. | Thank you for this comment/suggestion. The rationale behind the frequency of sample collection and follow-up periods has now been provided. | Lines 157-162 |
| Explain the selection of medical facilities for hiring. | Thank you for this comment/suggestion. An explanation has been provided on the selection of the medical facilities for this study. | Lines 130-131 |
| The Cary Blair medium is suitable for transporting *S. Typhi*, but to maintain sample integrity, storage conditions and maximum transit times should be considered. | Thank you for this comment/suggestion. Transport conditions have been described. | Lines 167-169 |
| Discuss whether AST made use of quality control measures other than reference strains? | Thank you for this comment/suggestion. QC measures taken have been described | Lines 203-204 |
| Justify the inclusion of antibiotics, especially third-generation cephalosporins. | Thank you for this comment/suggestion. All antibiotics included in this study all have action against gram negative bacteria and Cefotaxime which is a third generation cephalosporin is among the recommended drugs for treatment of typhoid fever in Kenya. | Lines 193-194 |
| Protocols for DNA extraction and sequencing are described in depth. But make it clear: (i) Read quality and depth coverage thresholds, (ii) How mistakes were reduced during assembly, (iii) MDR isolate selection criteria for sequencing. | Thank you for this comment/suggestion. The reads quality was assessed using FASTQ and the depth coverage was 50X. Adapters were trimmed before assembly and complete genomes assessed for quality using QUAST. MDR isolates for sequencing were randomly selected. | Line 223-225 |
| Discuss statistical techniques for comparing the prevalence of AMR in carriers and cases. Indicate the program used and any modifications made to account for possible confounders. | Thank you for this comment/suggestion. A description on the data analysis done and the software used has been included. No modification were made. | Lines 247-249 |
| Use the proper statistical metrics (such as p-values and confidence intervals) to quantify the observed variations in resistance between cases and carriers. | Thank you for this comment/suggestion. we have highlighted that P values were used to quantify observed variations. The findings are in the result section. | Lines 249-250 |
| Discuss genomic completeness criteria (such as N50) and the cutoff points for evaluating the quality of an assembly. | We have discussed the N50 genomic completeness criteria, and its significance in evaluating the quality of genome assemblies. In our study, the average N50 value was 197 kb, which is considered good for bacterial genome assemblies. Typically, an N50 above 50 kb is regarded as indicative of a high-quality assembly for bacteria, with longer N50 values suggesting even better contiguity. Our N50 value of 197 kb suggests that a significant portion of the genome is represented by relatively large contigs, which supports the reliability and completeness of the assembly. | Supplementary table |
| To confirm their authenticity, compare genome sizes and GC content to previously published data for *S. Typhi*. | Thank you for your suggestion. We have included a comparison of the genome sizes and GC content of the sequenced *S*. Typhi strains with previously published data. The genome sizes of our strains range from 4.7 to 4.9 Mb, with an average GC content of 52% which is consistent with the genome sequence of *S*. Typhi described in other studies. | Supplementary table |
| To confirm their authenticity, compare genome sizes and GC content to previously published data for *S. Typhi*. | Thank you for your suggestion. Data on comparison of genome sizes and GC content of this studys isolates to previously published data for S. Typhi has now been provided. | Supplementary table |
| Emphasize the importance of genotypes in connection to AMR (for example, 4.3.1.1 EA1 vs. 4.3.1.2 EA3). | Thank you for this comment/suggestion. From our findings, there was no connection between genotypes and AMR patterns observed. What is clear is that the reported Genotypes have been previously reported in Asia and Africa and the lineages and sub lineages have been reported in Eastern African Countries before.  The AMR genes present were related to first line antibiotics associated with MDR *S*. Typhi. | Discussion |
| Discuss why blaTEM-1D, catA1, and other AMR genes co-occur with *tetA* and *tetB*. | Thank you for this comment/suggestion. The co-occurrence of the above genes has now been explained. | Lines 408-409 |
| The table of genetic characteristics provides useful information. For a more thorough perspective, include summary data (such as the mean genome size). | Thank you for your suggestion. We have now included a summary of key genetic characteristics in the revised manuscript, including the mean genome size of the S. Typhi isolates. | Supplementary Table |
| Provide a summary table that compares the prevalence of AMR statistically between carriers and cases. | Thank you for this comment/suggestion. This table has now been provided in the results section | Lines 284-285 |
| It is important to interpret certain statistical findings, such as the lack of significance for the prevalence of the AMR gene between cases and carriers (e.g., limited sample size or uniform exposure). | Thank you for this comment/suggestion. This has been interpreted. | Lines 427-429 |
| Provide more details on novel discoveries, like genotypic variants unique to the Kenyan context, to support comparisons to earlier research. | Thank you for this comment/suggestion. More details on the genotypic variants unique to the Kenyan context, to support comparisons to earlier research have now been added. | Lines 410-420 |
| Explain how AMR trends may affect empirical treatment plans in Kenya? | Thank you for this comment/suggestion. The implication of empirical treatment plans to AMR in Kenya has been added on lines 385-387. | Lines 384-386 |
| Give specific suggestions for public health initiatives, including immunization drives or more stringent antibiotic stewardship in cities. | Thank you for this comment/suggestion. Specific public health initiates have been included in the discussion | Lines 446-448 |
| Although the discussion of bile-induced transcriptional alterations in carriers is intriguing, it may be extended to clarify how this supports the persistence of AMR. | Thank you for this comment/suggestion. As guided by reviewer 1, this information was deleted from the introduction, however a detailed description of AMR and carriers has been captured in the discussion. | Throughout the discussion |
| Add limitations such as the relatively small subset of sequenced isolates and potential sampling bias due to recruitment only from Mukuru. | Thank you for this comment/suggestion. Limitations have now been added on the manuscript as guided. | Lines 442-446 |
