# Supplementary material for: Genomic insights into the role of Salmonella Typhi carriers in antimicrobial resistance and typhoid transmission in Urban Kenya
Source: PLoS One. 2025 May 28;20(5):e0321879. doi: 10.1371/journal.pone.0321879 (PMC12118847; doi:10.1371/journal.pone.0321879)
Supplement: Attachment — Submitted filename: Reviewer Comments.pdf [file pone.0321879.s002.pdf]

The manuscript "Genomic analysis of *Salmonella Typhi* strains from typhoid patients and carriers reveals carriage as potentially important for community transmission of typhoid in Kenya" has been thoroughly examined. The study offers significant discoveries regarding the genomic characterisation of Salmonella Typhi and its connection to the spread of typhoid and antibiotic resistance. However, the manuscript needs to be revised. Here are some specific areas that require improvement:

Title and Abstract

- Take into consideration making the title more focused and specific. For example: "Genomic Insights into the Role of *Salmonella Typhi* Carriers in Antimicrobial Resistance and Typhoid Transmission in Urban Kenya."
- The focus on carriers and AMR profiles in the study should be clearly reflected in the title.
- The primary findings, including the frequency of genotypes and associated AMR patterns, should be outlined in the abstract. Public health recommendations should be included at the end.
- Give a one-sentence explanation of the methodology, highlighting AMR characterisation and whole genome sequencing.
- Do not repeat information (for example, resistance to first-line antibiotics is mentioned twice).

Introduction

- More quantitative information regarding the prevalence of typhoid fever in Kenya and its regional and worldwide distribution should be provided.
- Explain briefly why disease transmission is most prevalent in urban informal communities.
- Draw attention to inadequacies in earlier research, especially with relation to carriers' involvement in AMR transmission.
- Although the novelty and rationale are not stated clearly, describe how the study fills in knowledge gaps about the role of carriers in AMR.
- The introduction briefly mentions AMR-associated genotypes. Expound on why genotype 4.3.1 (H58) is significant in the Kenyan context.

Materials and Methods

- Although the longitudinal design is obvious, the rationale behind the frequency of sample collection and follow-up periods should be given.
- Explain the selection of medical facilities for hiring.
- The Cary Blair medium is suitable for transporting *S. Typhi*, but to maintain sample integrity, storage conditions and maximum transit times should be considered.
- Discuss whether AST made use of quality control measures other than reference strains?
- Justify the inclusion of antibiotics, especially third-generation cephalosporins.
- Protocols for DNA extraction and sequencing are described in depth. But make it clear: (i) Read quality and depth coverage thresholds, (ii) How mistakes were reduced during assembly, (iii) MDR isolate selection criteria for sequencing.
- Discuss statistical techniques for comparing the prevalence of AMR in carriers and cases. Indicate the program used and any modifications made to account for possible confounders.

Results

- Use the proper statistical metrics (such as p-values and confidence intervals) to quantify the observed variations in resistance between cases and carriers.
- Discuss genomic completeness criteria (such as N50) and the cutoff points for evaluating the quality of an assembly.
- To confirm their authenticity, compare genome sizes and GC content to previously published data for *S. Typhi*.
- Emphasize the importance of genotypes in connection to AMR (for example, 4.3.1.1 EA1 vs. 4.3.1.2 EA3).
- Discuss why blaTEM-1D, catA1, and other AMR genes co-occur with *tetA* and *tetB*.
- The table of genetic characteristics provides useful information. For a more thorough perspective, include summary data (such as the mean genome size).
- Provide a summary table that compares the prevalence of AMR statistically between carriers and cases.
- It is important to interpret certain statistical findings, such as the lack of significance for the prevalence of the AMR gene between cases and carriers (e.g., limited sample size or uniform exposure).

Discussion

- Provide more details on novel discoveries, like genotypic variants unique to the Kenyan context, to support comparisons to earlier research.
- Explain how AMR trends may affect empirical treatment plans in Kenya?
- Give specific suggestions for public health initiatives, including immunization drives or more stringent antibiotic stewardship in cities.
- Although the discussion of bile-induced transcriptional alterations in carriers is intriguing, it may be extended to clarify how this supports the persistence of AMR.
- Add limitations such as the relatively small subset of sequenced isolates and potential sampling bias due to recruitment only from Mukuru.
